# Enrollment and completion rates of a nationwide guided digital parenting program for children with disruptive behavior before and during COVID-19

**DOI:** 10.1007/s00787-024-02523-6

**Published:** 2024-08-14

**Authors:** Sakari Lintula, Andre Sourander, Susanna Hinkka-Yli-Salomäki, Terja Ristkari, Malin Kinnunen, Marjo Kurki, Altti Marjamäki, David Gyllenberg, Hyoun Kim, Amit Baumel

**Affiliations:** 1 Research Centre for Child Psychiatry, University of Turku, Lemminkäisenkatu 3, Teutori 3rd Floor, 20014 Turku, Finland; 2 INVEST Research Flagship Center, University of Turku, Turku, Finland; 3 Department of Child Psychiatry, Turku University Hospital, Turku, Finland; 4 ITLA Children’s Foundation, Helsinki, Finland; 5 Helsinki University Hospital, Helsinki, Finland; 6 National Institute for Health and Welfare, Helsinki, Finland; 7 Department of Child and Family Studies, Yonsei University, Seoul, South Korea; 8 Department of Community Mental Health, University of Haifa, Haifa, Israel

**Keywords:** Mental health, Public health, Parenting, Internet-based intervention, COVID-19 pandemic

## Abstract

Our aim was to study enrollment and completion levels for the internet-based and telephone-assisted Finnish Strongest Families Smart Website (SFSW) parent training intervention, for parents of young children with disruptive behavior before and after the COVID-19 lockdown period. Population-based screening was carried out on 39,251 children during routine check- ups at 4 years of age. The parents of children scoring at least 5 on the Strengths and Difficulties Questionnaire were assessed against inclusion and exclusion criteria. Associations with enrollment or completion were analyzed using logistic regression models. The effects of COVID-19 restrictions on these were estimated using interrupted timeseries analysis. Of 39,251 families, 4894 screened positive and met the eligibility criteria. Of those, 3068 (62.6%) decided to enroll in the SFSW program and 2672 (87.1%) of those families completed it. The highest level of disruptive behavior (OR 1.33, 95% CI 1.12–1.57, *p* < 0.001) and overall severity of difficulties (OR 2.22, 95% CI 1.91–2.57, *p* < 0.001) were independently associated with enrollment. Higher parental education was associated with enrollment and completion. Higher paternal age was associated with enrollment, and parent depressive symptoms with non-completion. The SFSW enrollment did not significantly change following the COVID-19 restrictions, while the completion rate increased (COVID-19 completion OR 1.75, 95% CI 1.22–2.50, *p* = 0.002). Guided digital parenting interventions increase the sustainability of services, by addressing the child mental health treatment gap and ensuring service consistency during crisis situations.

**Supplementary Information:**

The online version contains supplementary material available at 10.1007/s00787-024-02523-6.

## Introduction

A significant proportion of children with mental health problems do not receive any mental health services. This gap between the high prevalence of mental health disorders and low treatment utilization has been recognized as a matter of worldwide concern. To address the treatment gaps, it is important that evidence-based interventions are implemented in real life settings [1]. Parenting interventions have demonstrated efficacy in addressing disruptive behavior problems in children [2–5]. However, there are barriers to implementing parental training in public health settings [6]. Barriers to successful implementation include high cost, poor access, inconvenience, and low treatment fidelity [7, 8].

Internet-assisted, remote interventions have emerged as a way to reduce treatment gaps, because they offer affordable, accessible, and feasible services, and address challenges related to treatment fidelity [9]. Internet-assisted parent training interventions are not only effective, but also flexible and can overcome typical barriers to treatment, such as travelling, cost and the availability of nearby services [4, 5, 7, 10]. Successful implementation of digital interventions is related to enrollment and completion of the program. However, there is a notable problem with participation in digital mental health interventions in the real world [11, 12].

There is a lack of large-scale studies that examine enrollment and completion rates during the implementation of digital parent training programs. However, studies conducted in other settings on factors associated with enrollment and completion in digital parenting interventions, may provide valuable insights. Findings indicate that initial parenting confidence, parental agreement, internet usage, and program format could predict program completion, while demographic and clinical variables often do not [13–15]. Collectively, such studies underscore the multifaceted nature of engagement in digital parenting interventions and the necessity of addressing these factors to optimize program outreach through understanding of what works for whom [13–16].

This paper focuses on the Finnish Strongest Families Smart Website (SFSW) parenting program [10, 17, 18], which has been implemented nationwide in the Finnish primary healthcare system. Four-year-old children are screened during the free child healthcare checkups that are offered to all parents. The target group for the intervention is the parents of those children with disruptive behaviors, such as temper tantrums, oppositional behavior and aggression. It has previously been reported that the SFSW program has a lasting and positive effect on decreasing child behavior problems during a randomized controlled trial and implementation studies [4, 5, 19, 20].

This study examined two distinct topics relating to overall participation in the implementation of SFSW: enrollment and completion. The first aim of the study was to examine program enrollment and completion rates. The enrollment rate was defined as the proportion of families who fulfilled the screening criteria for the targeted intervention, and chose to participate. The completion rate was defined as the proportion of families who completed the whole program. The second aim was to explore demographics, parenting styles, parent and child psychopathology and parent perceptions of the severity of the child’s problems associated with enrollment or completion. Finally, since during the COVID-19 pandemic interruptions occurred in other nationwide services [21], we tested if interruptions in enrollment and completion rates occurred in SFSW.

## Methods

### Intervention and implementation

The SFSW parenting intervention program is aimed at the parents of 4-year-old children with disruptive behavior difficulties [10]. Parents are provided with a password-controlled interactive website that provides them with a series of e-learning modules with psychoeducative reading material, audio and video examples and home assignments, and they also receive weekly calls from a trained coach [17]. The coaches are health care professionals with special training for this intervention, including education about conduct problems, parent training and digital interventions. The coaches attended weekly group case conferences led by a coach supervisor, made self-assessments after each call and got regular feedback from their supervisor.

The program is designed to teach the parents the skills that they can use to cope with difficult situations in their everyday lives and to form a close relationship with their child. The need to practice these techniques with their child between sessions and the ongoing objectives are individually tailored to the parent and child during the weekly coaching calls. The SFSW program is based on positive parenting, social learning theory and cognitive behavioral therapy and consists of 10 weekly sessions. It includes seven weeks of basic skills covering positive, active, and self-controlled parenting and three weeks on enhancing and generalizing skills. There is an optional 11th booster session after the program has been completed. Mean duration of weekly calls was 37.3 (SD 11.0) minutes and mean duration of weekly website access was 45.3 (SD 19.3) minutes, respectively. The average total duration of the program is therefore 13.8 (SD 4.3) hours. Details of the weekly session are shown in Table S1.

By summer 2021, the SFSW program had been implemented in 99/309 (32%) of the Finnish municipalities and that was the end point chosen for this study. We included all families who were invited to take part in the program from the start of its implementation on January 1, 2015 until June 30, 2021. This ensured that every participant included in this report would have either rejected the invitation to take part, completed the program or not completed the program. The implementation of the program included constant collaboration with respected referral systems and ongoing training for local public health nurses who took care of screening procedures at local child health clinics.

Screening was conducted at the standard 4-year-old healthcare checkup by trained nurses using the conduct problems subscale of the Strengths and Difficulties Questionnaire (SDQ). The parents were also sent a questionnaire asking for demographic information one month before the scheduled checkup and asked to bring the questionnaire to the checkup appointment. The questionnaires were then sent to the research center, along with the SDQ screening results [17].

The inclusion criteria for being invited to take part in the SFSW were two-fold. The first was a score of 5 or higher on the SDQ Conduct Scale, which corresponded to an 80th percentile cut-off point. The second was that the parents reported that the child has at least minor difficulties, based on the SDQ supplementary item that asked about the severity of the overall problems. Our exclusion criteria were that the child was not able to speak in full sentences and that at least one parent was taking part in another intervention. We also excluded children who met the diagnostic criteria for autism or pervasive development disorder, Down syndrome, fetal alcohol syndrome, mental retardation, or a genetic diagnosis that would lead to mental retardation.

The recruitment and enrollment process started at the child health center. The nurses encouraged the parents to participate in the program if the SDQ screening process was positive. If the child met the SDQ inclusion criteria, the parents received a phone call from SFSW recruitment staff, who confirmed that the exclusion criteria did not apply. They then introduced the parents to the SFSW. Confirmed enrollment was defined as when the family was deemed eligible and provided their formal consent on the intervention website. The intervention started with an introductory call to the parents by a coach.

Our key priorities included engagement and completion*.* To improve engagement, reminders were sent to parents who stopped interacting with their individual learning program. This started with an automated message and was followed up by a phone call from the coach within a week if the parents remained inactive. The weekly coaching phone calls were scheduled when the parents had free time and were most receptive to the program content. During these calls the coaches provided constant positive feedback about the parents’ use of skills in order to keep them focused and motivated. This also emphasized the importance of parents providing positive feedback to their children. The parents needed to continue with the individual learning on the website to receive the coaching calls.

### Measures and variables

The primary outcomes were enrollment to the program and program completion. We also included questions about demographics, parent reports of child psychopathology and perceived difficulties, parent psychopathology, and parenting styles.

Enrollment was defined as accepting the invitation to take part in the intervention after an introductory phone call from a member of the research team. Families that enrolled in the intervention and completed at least 10 weekly sessions were defined as completers. The 11th session was an optional booster call after the program ended to discuss how the goals of the program had been achieved, and that is why it was not a criterion for completion.

Three subscales of the validated Finnish version of the SDQ [22] were used to assess child psychopathology: conduct problems, emotional symptoms, and hyperactivity/inattention. Each subscale was measured by 5 distinct items, with three possible responses the caregiver is using to describe the child’s behavior: not true, somewhat true, and certainly true (minimum score for each subscale was 0 and maximum score 10). The conduct problem scale was categorized into three groups (scoring 5, 6–7 and above 7). These represented the 80th, 90th and 95th percentile cutoffs for the study sample, respectively. The emotional symptoms scale was dichotomized at the 90th percentile as 2 or less and 3 or more. The hyperactivity/inattention scale was similarly dichotomized at the 90th percentile as 6 or less and 7 or more. An item asking about the level of the child’s overall difficulties was also included in the SDQ supplement. It asked whether the children had overall difficulties in one or more of the following areas: emotions, concentration, behavior, or being able to get along with people. Our study used three reported categories: no, minor, and more severe difficulties. The SDQ supplement also asked how long the child’s difficulties had been present and these were categorized as less than 1 year vs. 1 year or more.

The 21-item Depression Anxiety Stress Scale (DASS-21) [23] was used to measure parental depression, anxiety and stress. Each of the three DASS-21 subscales, depression, anxiety, and stress, has seven items. Each item has four possible responses ranging from “does not apply to me at all” to “apply to me very much or most of the time” (minimum score for each subscale is 0 and maximum score is 42).

The 30-item Parenting Scale (PS), revised by Rhoades & O’Leary [24] and used in previous Strongest Families studies [10] measuring parenting styles was used. PS has three subscales: laxness and over-reactivity each consist of five items and hostility that consists of three items. Each item is rated using a 7-point scale, covering the spectrum between two opposites (minimum score for each subscale is 1 and maximum score is 7).

We felt it was important to take account of the national COVID-19 restrictions in Finland. To do this, we created separate dummy variables appointing indicating times: before the national pandemic lockdown on March 16 2020, and during the restrictions from that date until June 30 2021. Lastly, the monthly count for families enrolling into the program was used to see if there were any sudden changes in the numbers enrolling in the program.

### Statistical analysis

Logistic regression analyses were conducted to analyze enrollment and completion odds. We analyzed which explanatory variables increase the odds of enrollment and completion separately. Univariate logistic regressions were performed and all significant variables (*p*-value < 0.05) were included in the multivariate logistic regressions. All multivariate models were adjusted for the child’s sex.

The effects of the COVID-19 pandemic restrictions on enrollment rates were inspected with an interrupted time series analysis [25]. We fitted linear time trends into the time series regressions and included the separate dummy variable outlined above to represent Finnish COVID-19 restrictions after March 16, 2020. Step change models were fitted [25] to inspect if there was a change in enrollment. In addition, we included calendar month as a categorical predictor in the models to adjust for seasonal effects [25]. We also inspected if there was a drop in the number of parental interventions that started in 2020 or from January to June 2021 to see if there was any possible higher selection bias at any point.

All continuous variables were standardized to 0 mean and unit variance. Odds-Ratios (OR), 95% CI and *p*-value are reported for all analyses. Statistical analysis was conducted with R-software version 4.3.0 [26].

## Results

**Fig. 1:**
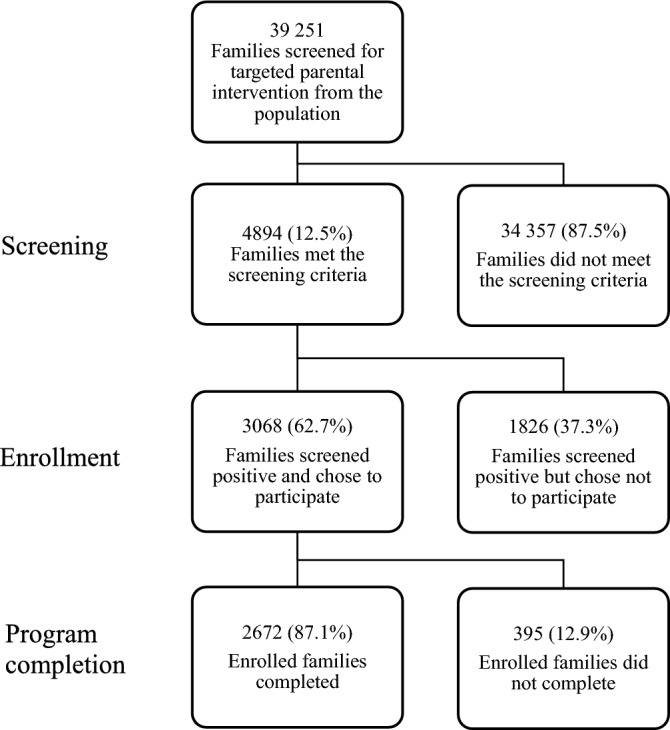
Flowchart of sampling and study design

The flow chart of screening, enrollment and completion is presented in Fig. 1. Altogether 39,251 children were screened for disruptive behavior and 4894 families (12.3%) met the eligibility criteria for the intervention.

### Characteristics associated with child disruptive behaviors

Table 1 presents the demographic and child psychopathology variables of the families who fulfilled the criteria of having disruptive behavior when screened for the targeted parent training intervention. Multivariate analyses that included demographic variables showed that being screened positive was independently associated with the child being male rather than female (OR 1.62, 95% CI 1.52–1.73, *p* < 0.001) and living in another household structure that did not include both biological parents (OR 1.36, 95% CI 1.23–1.50, *p* < 0.001). Mothers’ age of 35 years or above was associated with a smaller chance of being screen positive in comparison to 25–35 years of age (OR 0.91, 95% CI 0.84–0.98, *p* = 0.014). Lower paternal education was associated with being screened positive but this did not remain significant when the data were controlled with other variables in the multivariate analyzes.

**Table 1:** Demographics, and child psychopathology of the 39 251 families of children screened at 4-years of age for the targeted SFSW parent training intervention

	Screened *n*	Screened positive^a^ *n* (%)	Univariate		Multivariate^n^	
			OR (95% CI)^b^	*p* value	OR (95% CI)^b^	*p* value
*Demographic variables*						
Child's sex^c^						
Female	19,197	1878 (9.8%)	Ref	–	Ref	–
Male	19,895	2996 (15.1%)	1.63 (1.54, 1.74)	< 0.001	1.62 (1.52, 1.73)	< 0.001
Maternal age (years)^d^						
< 25	863	123 (14.3%)	1.10 (0.90, 1.33)	0.353	0.95 (0.75, 1.22)	0.696
25–35	21,183	2788 (13.2%)	Ref	–	Ref	–
> 35	16,848	1937 (11.5%)	0.86 (0.81, 0.91)	< 0.001	0.91 (0.84, 0.98)	0.014
Paternal age (years)^e^						
< 25	337	55 (16.3%)	1.27 (0.94, 1.70)	0.114	1.16 (0.82, 1.65)	0.392
25–35	15,548	2077 (13.4%)	Ref	–	Ref	–
> 35	21,693	2516 (11.6%)	0.85 (0.80, 0.91)	< 0.001	0.93 (0.86, 1.00)	0.058
Maternal education^f^						
College or university degree	22,996	2845 (12.4%)	Ref	–		
Vocational or high school	14,283	1834 (12.8%)	1.04 (0.98, 1.11)	0.184		
Obligatory school education	1536	185 (12.0%)	0.97 (0.83, 1.14)	0.706		
Paternal education^g^						
College or university degree	16,929	1986 (11.7%)	Ref	–	Ref	–
Vocational or high school	17,852	2296 (12.9%)	1.11 (1.04, 1.18)	0.001	1.04 (0.97, 1.11)	0.226
Obligatory school education	2066	284 (13.7%)	1.20 (1.05, 1.37)	0.008	1.08 (0.94, 1.25)	0.255
Family structure^h^						
Two biological parents	33,635	4010 (11.9%)	Ref	–		
Other	5451	867 (15.9%)	1.40 (1.29, 1.51)	< 0.001	1.36 (1.23, 1.50)	< 0.001
Overall difficulties^i,m^						
No difficulties	20,924	0	NA			
Minor difficulties	14,078	2548 (18.1%)	Ref	–		
Major difficulties	4131	2346 (56.8%)	5.95 (5.52, 6.41)	< 0.001		
SDQ Conduct scale^j,m^						
< 80th percentile	31 336	0 (0%)	NA			
80–90th	3 847	1999 (52.0%)	Ref	–		
90–95th	2 119	1378 (65.0%)	1.72 (1.54, 1.92)	< .001		
≥ 95th	1 949	1517 (77.8%)	3.25 (2.87, 3.68)	< 0.001		
SDQ hyperactivity scale^k,n^						
< 90th percentile	34 723	2977 (8.6%)	Ref	–		
≥ 90th	4528	1917 (42.3%)	7.83 (7.30, 8.40)	< 0.001		
SDQ emotional problems scale^l,n^						
< 90th percentile	34 870	3645 (10.5%)	Ref	–		
≥ 90th	4 381	1249 (28.5%)	3.42 (3.17, 3.68)	< 0.001		

^a^Screened positive using SDQ. ^b^Odds ratio (OR) and confidence interval (CI). ^c^Missing = 159. ^d^Missing = 357. ^e^Missing = 1 673. ^f^Missing = 436. ^g^Missing = 2 404. ^h^Missing = 165. ^i^Missing = 118. ^j^Missing = 0. ^k^Missing = 0. ^l^Missing = 0. ^m^80th Percentile in conduct scale and at least minor difficulties were screening criteria. ^n^Child psychopathology variables were not included in the multivariate analyses since they were included partly in screening criteria

Psychopathology variables were not included in the multivariate analysis, as child disruptive behavior was part of screening criteria. All psychopathology measures were strongly associated with being screened positive in the univariate analyzes.

### Characteristics associated with enrollment

The percentage of families who were invited to take part and actually enrolled in the program was 62.7% (95% CI 62.7–64.1%). As shown in Table 2 multivariate analyzes, enrollment was associated with the highest level (> 95th percentile cutoff point) of child’s disruptive behavior (OR 1.33, 95% CI 1.12–1.57, *p* < 0.001), major severe difficulties (OR 2.22, 95% CI 1.91–2.57, *p* < 0.001) and long- term difficulties (OR 1.31, 95% CI 1.14–1.51, *p* < 0.001). Comorbid emotional problems (> 90th percentile cutoff point) were also associated with enrollment (OR 1.25, 95% CI 1.07–1.46, *p* = 0.006). The results remained the same when child psychopathology measures were analyzed as continuous variables (Table S2). However, lower maternal and paternal education were associated with non-enrollment, as shown in Table 2. Younger paternal age was associated with non-enrollment.

**Table 2:** Demographics, and child psychopathology for the 4894 families who screened positive and the 3068 who chose to participate in the targeted parental training program

	Screened positive	Enrolled^a^	Univariate		Multivariate	
	*n*	*n* (%)^a^	OR (95% CI)^b^	*p* value	OR (95% CI)^b^	*p* value
*Demographic variables*						
Child's sex^c^						
Male	2996	1934 (64.6)	Ref	–	Ref	–
Female	1878	1121 (59.7)	0.81 (0.72, 0.92)	< 0.001	0.92 (0.81, 1.06)	0.247
Maternal age (years)^d^						
< 25	123	59 (48.0)	0.60 (0.42, 0.86)	0.005	0.90 (0.57, 1.43)	0.648
25–35	2788	1691 (60.7)	Ref	–	Ref	–
> 35	1937	1294 (66.8)	1.31 (1.16, 1.47)	< 0.001	0.96 (0.81, 1.13)	0.608
Paternal age (years)^e^						
< 25	55	22 (40.0)	0.47 (0.27, 0.81)	0.006	0.46 (0.24, 0.90)	0.024
25–35	2077	1221 (58.8)	Ref	–	Ref	–
> 35	2516	1687 (67.1)	1.43 (1.26, 1.61)	< 0.001	1.21 (1.03, 1.42)	0.019
Maternal education^f^						
College or university degree	2845	1979 (69.6)	Ref	–	Ref	–
Vocational or high school	1834	996 (54.3)	0.52 (0.46, 0.59)	< 0.001	0.61 (0.52, 0.71)	< 0.001
Obligatory school education	185	77 (41.6)	0.31 (0.23, 0.42)	< 0.001	0.36 (0.25, 0.54)	< 0.001
Paternal education^g^						
College or university degree	1988	1399 (70.4)	Ref	–	Ref	–
Vocational or high school	2296	1331 (58.0)	0.58 (0.51, 0.66)	< 0.001	0.74 (0.63, 0.85)	< 0.001
Comprehensive school	284	153 (53.9)	0.49 (0.38, 0.63)	< 0.001	0.64 (0.48, 0.85)	< 0.001
Family structure^h^						
Two biological parents	4010	2536 (63.2)	Ref	–		
Other	867	521 (60.1)	0.88 (0.75, 1.02)	0.082		
*Child psychopathology*						
Overall difficulties^i^						
Minor difficulties	2548	1347 (52.9)	Ref	–	Ref	–
Major difficulties	2346	1721 (73.4)	2.46 (2.18, 2.77)	< 0.001	2.22 (1.91, 2.57)	< 0.001
Length of difficulties^j^						
Less than 1 year	2985	1733 (58.1)	Ref	–	Ref	–
One year or more	1807	1279 (70.8)	1.75 (1.54, 1.98)	< 0.001	1.31 (1.14, 1.51)	< 0.001
SDQ Conduct scale^k^						
80–90th percentile	1999	1142 (57.1)	Ref	–	Ref	–
90–95th	1378	843 (61.2)	1.18 (1.03, 1.36)	0.019	1.02 (0.87, 1.20)	0.795
≥ 95th	1517	1083 (71.4)	1.87 (1.62, 2.16)	< 0.001	1.33 (1.12, 1.57)	0.001
SDQ Hyperactivity scale^l^						
< 90th percentile	2977	1777 (59.7)	Ref	–	Ref	–
≥ 90th	1917	1291 (67.3)	1.39 (1.23, 1.57)	< 0.001	1.10 (0.95, 1.28)	0.197
SDQ Emotional problems scale^m^						
< 90th percentile	3645	2201 (60.4)	Ref	–	Ref	–
≥ 90th	1249	867 (69.4)	1.49 (1.30, 1.71)	< 0.001	1.25 (1.07, 1.46)	0.006

^a^Amount and proportion of families who chose to participate. ^b^Odds ratio (OR) and confidence interval (CI). ^c^Missing = 20. ^d^Missing = 46. ^e^Missing = 246. ^f^Missing = 30. ^g^Missing = 328. ^h^Missing = 17. ^i^Missing = 0. ^j^Missing = 102. ^k^Missing = 0. ^l^Missing = 0. ^m^Missing = 0

### Characteristics associated with completion

Of those who enrolled, 87.1% (95% CI 85.9–88.3%) completed the whole program, and 89.1% (95% CI 88.0–90.2%) completed all of the seven basic skills training sessions. Families who did not complete the intervention had a median of three training sessions (mean 3.5, standard deviation 2.6).

As shown in Table 3, child psychopathology was not associated with program completion. Parents with depressive symptoms were less likely to have completed the program (OR 0.83, 95% CI 0.72–0.97). Lower maternal and paternal education were independently associated with non-completion. More than 80% of the parents with vocational or high school education completed the program. Among parents who had completed only the obligatory 9 years of schooling, 64.9% of mothers and 77.1% of fathers finished the program.

**Table 3:** Demographics, and child psychopathology for the 3068 enrolled families and the 2672 who completed the parental training program for 4-year-old children

	Enrolled	Completers^a^	Univariate		Multivariate	
	*n*	*n* (%)^a^	OR (95% CI)^b^	*p* value	OR (95% CI)^b^	*p* value
*Demographic variables*						
Child’s sex^c^						
Male	1934	1687 (87.2)	Ref	**–**	Ref	**–**
Female	1121	974 (86.9)	0.97 (0.78, 1.21)	0.785	0.95 (0.74, 1.20)	0.653
Maternal age (years)^d^						
< 25	59	42 (71.2)	0.39 (0.22, 0.70)	0.002	0.61 (0.30, 1.26)	0.181
25–35	1691	1460 (86.3)	Ref	–	Ref	–
> 35	1294	1153 (89.1)	1.29 (1.03, 1.62)	0.024	1.06 (0.79, 1.42)	0.704
Paternal age (years)^e^						
< 25	22	18 (81.8)	0.73 (0.25, 2.19)	0.578	1.33 (0.38, 4.71)	0.655
25–35	1221	1050 (86.0)	Ref	–	Ref	–
> 35	1687	1503 (89.1)	1.33 (1.06, 1.66)	0.012	1.02 (0.77, 1.35)	0.894
Maternal education^f^						
College or university degree	1979	1790 (90.4)	Ref	**–**	Ref	**–**
Vocational or high school	996	821 (82.4)	0.50 (0.40, 0.62)	< 0.001	0.69 (0.52, 0.91)	0.009
Obligatory school education	77	50 (64.9)	0.20 (0.12, 0.32)	< 0.001	0.27 (0.15, 0.50)	< 0.001
Paternal education^g^						
College or university degree	1399	1280 (91.5)	Ref	**–**	Ref	**–**
Vocational or high school	1331	1133 (85.1)	0.53 (0.42, 0.68)	< 0.001	0.67 (0.51, 0.89)	0.005
Obligatory school education	153	118 (77.1)	0.31 (0.21, 0.48)	< 0.001	0.50 (0.31, 0.81)	0.005
Family structure^h^						
Two biological parents	2536	2239 (88.3)	Ref	**–**	Ref	–
Other	521	425 (81.6)	0.59 (0.46, 0.76)	< 0.001	1.08 (0.77, 1.51)	0.659
Child psychopathology						
Overall difficulties^i^						
Minor difficulties	1347	1171 (86.9)	Ref	–		
Major difficulties	1721	1502 (87.3)	1.03 (0.83, 1.28)	0.779		
Length of difficulties^j^						
Less than 1 year	1733	1503 (86.7)	Ref	–		
One year or more	1279	1126 (888.0)	1.13 (0.91, 1.40)	0.287		
SDQ Conduct scale^k^						
80–90th percentile	1142	1007 (88.2)	Ref	–		
90–95th	843	730 (86.6)	0.87 (0.66, 1.13)	0.292		
> 95th	1083	936 (86.4)	0.85 (0.66, 1.10)	0.215		
SDQ Hyperactivity scale^l^						
< 90th percentile	1777	1570 (88.4)	Ref	–	Ref	–
> 90th	1291	1103 (85.4)	0.77 (0.63, 0.96)	0.018	1.03 (0.81, 1.32)	0.792
SDQ Emotional problems scale^m^						
< 90th percentile	2201	1930 (87.7)	Ref	–		
> 90th	867	743 (85.7)	0.84 (0.67, 1.06)	0.139		
	Mean (SD)	Mean (SD)	OR (95% CI)^a,b^	*p* value	OR (95% CI)^a,b^	*p* value
Parent psychopathology (DASS)						
Depression^n^	5.6 (6.5)	5.4 (6.3)	0.80 (0.73, 0.87)	< 0.001	0.83 (0.72, 0.97)	0.016
Anxiety^n^	3.1 (4.5)	2.9 (4.3)	0.83 (0.76, 0.91)	< 0.001	1.02 (0.89, 1.17)	0.761
Stress^n^	11.6 (7.3)	11.4 (7.2)	0.83 (0.75, 0.92)	< 0.001	1.00 (0.84, 1.17)	0.965
Parenting (PS)						
Laxness^o^	2.7 (0.8)	2.7 (0.8)	0.94 (0.85, 1.04)	0.242		
Hostility^o^	1.8 (0.8)	1.8 (0.8)	0.83 (0.75, 0.92)	< 0.001	0.90 (0.80, 1.01)	0.080
Over-reactivity^o^	3.8 (1.0)	3.8 (1.0)	1.08 (0.97, 1.20)	0.171		

^a^Amount and proportion of families who completed the program. ^b^Odds ratio (OR) and confidence interval (CI). ^c^Missing = 13. ^d^Missing = 24. ^e^Missing = 138. ^f^Missing = 16. ^g^Missing = 185. ^h^Missing = 11. ^i^Missing = 0. ^j^Missing = 56. ^k^Missing = 0. ^l^Missing = 0. ^m^Missing = 0. ^n^Missing = 2. ^o^Missing = 2. ^p^OR is per 1 SD change

### The impact of COVID-19 restrictions on enrollment and completion

**Fig. 2:**
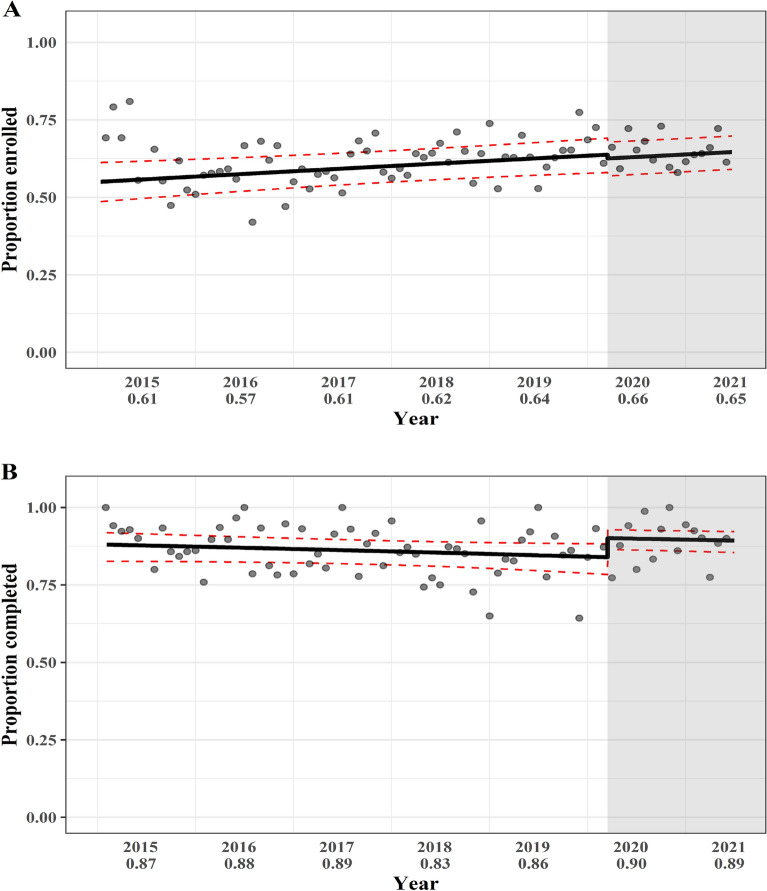
**A** Enrollment rate from January 1 2015 to June 30 2021. Solid line shows estimated enrollment rate and the estimated change on March 16 2020. Red dashed lines are 95% CIs. Numbers within brackets are annual enrollment rates. **B** Completion rate from January 1 2015 to June 30 2021. Shaded grey area indicates time after COVID-19 restrictions. Points are monthly enrollment and completion rates. Numbers below years are annual enrollment and completion proportions

Program enrollment and completion rates based on pre- and during-COVID-19 restrictions time are presented in Fig. 2. There was no statistically significant step-change in the enrollment rate at the onset of COVID-19 restrictions (OR for enrollment: 0.95, 95% CI 0.78–1.16, *p* = 0.60), although a linear increase over time in enrollment was estimated (OR for enrollment per year: 1.08, 95% CI 1.02–1.12, *p* = 0.005). However, for completing the program, a statistically significant increase in completion rate was estimated at the onset of COVID-19 restrictions (OR for completion step-change: 1.75, 95% CI 1.22–2.50, *p* = 0.002); the linear underlying trend for completion was not statistically significant (OR 0.93, 95% CI 0.85–1.02, *p* = 0.15); (Table S3).

## Discussion

This study reported key elements in the transition from evidence-based psychosocial interventions to implementation in real-world clinical practice. Our study findings provide important guidance for organizations and stakeholders planning early, population-based interventions for preschool children who face a high risk of later adversities.

The SFSW program is unique because it is based on screening children from the general population during routine primary health checkups at 4 years of age. Children who screened positive were more likely to be males, did not live in households with both of their biological parents and had less educated fathers. They also had more severe and comorbid psychopathology. These findings were in line with previous research about risk factors associated with disruptive behavior and comorbidity [27].

The program enrollment rate was 62.7% and completion rate was 87.1%. The enrollment rate of the parents of children with the highest level of disruptive behavior was 71.4%. These findings suggest that the SFSW implementation may reach children with the highest risk for developing adversities later in development [28, 29]. We are not aware of studies presenting enrollment and completion rates of a similar program under nationwide implementation. However, we could examine our metrics in relation to resembling programs that were examined under trial settings. A systematic review examining enrollment data across more than 50 studies of behavioral parent training programs report enrollment rates that are not higher than 75% [30]. While these rates are lower than the one we reported in this paper, we note that it is difficult to make such comparisons as enrollment in a study is influenced by factors related to the trial being conducted. In terms of completion rates, different studies examining the usage of digital parent training programs report a mean percentage of completed sessions around 70% [7, 31]. Therefore, the 87% completion rate shown in the present study can be considered exceptionally high.

Lower parental education and younger paternal age were associated with not enrolling in the program, while lower parental education and parental psychopathology were associated with not completing it. Additionally, parental depression independently lowered completion rates, in line with previous research [32, 33]. Our study found that more parents with vocational or high school education completed the program than those who had only completed the obligatory 9 years of school education. Other studies have found that parents with lower education levels were associated with a higher probability of non-participation [30, 34].

The present study covered the implementation of the program before and during part of the COVD-19 pandemic. There is a need for society to learn how mental health support systems work during crises [35]. Our results indicate that program enrollment or completion did not decrease during the pandemic. In fact, the completion rate increased at the start of the Finnish COVID-19 restrictions. Our findings support the assumption that remote, digitally-based interventions that have already been implemented may be able to increase the sustainability of services during crises.

The high enrollment and completion rates were probably due to the complementary components of the program, namely the phone coaching, psychoeducational Internet-based material and the accessible and convenient remote delivery of the intervention. The context of the program was well-defined, including the clear definition of the target population and the well-defined inclusion and exclusion criteria. The program also had a clear structure, including the description of the core components, which was practiced through modelling, practice, feedback, and support. Fidelity to the intervention was ensured by the implementation phase, the way the program was conducted, and the telephone coaching was centralized and supervised, and the systematic quality assurance provided by the digital platform. The well-trained coaches received constant support and feedback and formed good relationships with the parents [17], which is central to the success of any intervention [36]. The remote delivery of the program was much easier for the parents than face-to-face interventions, because they did not need to leave home or work or make childcare arrangements. These benefits were particularly evident during the COVID-19 restrictions. It is likely that all these factors motivated the parents to complete the intervention.

It has previously been emphasized that a solid framework and a structured implementation plan are needed for the successful transition from evidence-based psychosocial interventions to real-world clinical practice [37]. We systematically followed a structured plan during the implementation process [17]. The SFSW program contained core implementation drivers that facilitated the process when the intervention was implemented in the primary healthcare settings. In addition, the program was effectively administered by holding regular meetings with the directors of child and family services and providing them with user-friendly reports. Media coverage also raised awareness of the program, which made it easier to recruit families and increased the perceived value of the program [17]. We believe that this is one explanation for the rising trend in the enrollment rate. In fact, all of these implementation components probably played a crucial role in enrolling parents in the program.

## Limitations

There were some limitations to our study. Only parental reports of child behavior were used in the analyses. It would have been helpful to validate these reports through direct observations of their parenting, clinical observations, or teacher ratings. Qualitative information from interviews with healthcare personnel and parents might have provided more information about the reasons for not enrolling in the program and not completing it. Unfortunately, we did not collect detailed information about reasons for non-enrollment or non-completion.

## Conclusions

This study shows that the SFSW program resulted in high enrollment and completion rates. There is a huge worldwide need to provide parental training, and only a small proportion of those who need it actually receive evidence-based treatment for their children’s disruptive behavioral problems. Using Internet technology to move interventions outside traditional clinics and into people’s homes can provide better access to mental health services. Web-based interventions can also remove the barriers associated with face-to-face interventions. However, there is a challenge with getting parents with lower education levels or psychiatric morbidity to participate in such programs.

The enrollment and completion rates did not decrease during Finland’s COVID-19 restrictions. This emphasizes the importance of providing remote interventions to increase the sustainability of child mental health services during global crises, such as new pandemics, wars, and environmental catastrophes.

## Supplementary Information

Below is the link to the electronic supplementary material.

Supplementary file1 (DOCX 86 KB)

## Data Availability

No datasets were generated or analysed during the current study.
