# Supplementary material for: Enrollment and completion rates of a nationwide guided digital parenting program for children with disruptive behavior before and during COVID-19
Source: Eur Child Adolesc Psychiatry. 2024 Aug 14;34(2):739–49. doi: 10.1007/s00787-024-02523-6 (PMC11868137; doi:10.1007/s00787-024-02523-6)
Supplement: Supplementary file 1 — Supplementary file1 (DOCX 86 KB) [file 787_2024_2523_MOESM1_ESM.docx]

**Supplementary material**

*Table S1.* The content and key elements of the Strongest Families Smart Website (SFSW) parent training intervention

| **Session** | **Key training elements** | **Parental goals** | **Coaching elements** |
| --- | --- | --- | --- |
| **Introduction to the program** | Set up the parents for success | Reorient the parents How to break the negative circle | Working alliance  Identifying behavior problems  Goal setting  Present the first weekly theme |
| **Notice the good** | Positive and active parenting | Boost self-esteem of the child and parents and change the parents’ views of the child | Working alliance  Evaluate the goal setting by modeling, practice, such as role play, feedback, support |
| **Spread attention around** | Positive, impartial parenting | Strengthen child’s empathy skills | Same as above |
| **Ignore whining and complaining** | Positive, self-controlled parenting | Teaches parents self-regulation | Same as above |
| **Prepare for changes** | Positive, proactive parenting | Reinforce good daily routines | Same as above |
| **Plan ahead at home** | Positive, proactive parenting | Reinforce child’s active role and involve them in planning | Same as above |
| **Reinforce by rewarding** | Positive, active parenting | Involve the child in planning and reinforce good daily routines | Same as above |
| **Plan ahead outside the home** | Positive, proactive parenting | Reinforce child’s active role and involve them in planning | Same as above |
| **Cooperate with daycare** | Positive cooperation and communication between parent and day care | Help child to manage and succeed | Same as above |
| **Plan how to use time-out** | Positive, self-controlled parenting | Teach self-regulation and consistency | Reassure and use positive skills  How to use time-out |
| **Revise -**  **Problem solving and future application of skills** | Positive daily parenting in future | Remind parents of positive, proactive parenting skills | Ensure that parent is using all the skills and stays on track. |

*Table S2.* Analysis using continuous psychopathology IVs.

|  | Univariate | |  | Multivariate^b^ | |
| --- | --- | --- | --- | --- | --- |
|  | OR^a^ (95% CI) | *P* value |  | OR^a^ (95% CI) | *P* value |
|  | Enrolment | | | | |
| Conduct scale | 0.75 (0.70, 0.79) | <.001 |  | 0.87 (0.80, 0.94) | <.001 |
| Hyperactivity scale | 0.84 (0.79, 0.89) | <.001 |  | 0.93 (0.87, 1.01) | .082 |
| Emotional scale | 0.81 (0.76, 0.86) | <.001 |  | 0.88 (0.82, 0.95) | <.001 |
|  | Completion | | | | |
| Conduct scale | 1.05 (0.95, 1.16) | .348 |  | 1.01 (0.90, 1.14) | .820 |
| Hyperactivity scale | 1.13 (1.02, 1.26) | .020 |  | 0.94 (0.83, 1.07) | .358 |
| Emotional scale | 1.11 (1.01, 1.22) | .037 |  | 1.07 (0.95, 1.19) | .259 |
| ^a^ Variables were standardized so that OR is change per 1 SD.  ^b^ Multivariate analyses included all variables, as included in analyses using categorical SDQ subscales. | | | | | |

*Table S3.* Associations between time and COVID-19 restriction onset, and enrolment and completion in remote parental intervention.

|  | OR (95% CI)^a^ | *P* value |
| --- | --- | --- |
| **Enrolment** | | |
| Linear time (1 year) | 1.08 (1.02, 1.12) | .005 |
| Step-change^b^ | 0.95 (0.78, 1.16) | .60 |
| Month |  |  |
| January | Ref. | - |
| February | 0.96 (0.74, 1.27) | .79 |
| March | 0.94 (0.71, 1.25) | .69 |
| April | 1.05 (0.79, 1.39) | .73 |
| May | 1.11 (0.85, 1.45) | .44 |
| June | 1.01 (0.77, 1.32) | .96 |
| July | 0.90 (0.65, 1.25) | .52 |
| August | 0.95 (0.74, 1.25) | .74 |
| September | 1.11 (0.85, 1.47) | .44 |
| October | 1.08 (0.82, 1.41) | .59 |
| November | 1.06 (0.79, 1.43) | .68 |
| December | 0.99 (0.72, 1.35) | .94 |
| **Completion** | | |
| Linear time (1 year) | 0.93 (0.85, 1.02) | .15 |
| Step-change^b^ | 1.75 (1.22, 2.50) | .002 |
| Month |  |  |
| January | Ref. | - |
| February | 1.09 (0.65, 1.82) | .74 |
| March | 0.92 (0.55, 1.52) | .74 |
| April | 0.67 (0.39, 1.14) | .14 |
| May | 0.82 (0.48, 1.37) | .45 |
| June | 1.06 (0.61, 1.85) | .82 |
| July | 2.38 (0.66, 14.29) | .26 |
| August | 0.97 (0.57, 1.61) | .90 |
| September | 0.97 (0.54, 1.75) | .92 |
| October | 0.93 (0.56, 1.52) | .77 |
| November | 1.12 (0.62, 2.04) | .71 |
| December | 0.93 (0.52, 1.72) | .84 |
| ^a^ Odds ratio and confidence interval.  ^b^ Estimated change on March 16^th^ 2020, when COVID-19 restrictions were put in place. | | |
